# The Use of Gel Electrophoresis to Separate Multiplex Polymerase Chain Reaction Amplicons Allows for the Easy Identification and Assessment of the Spread of Toxigenic *Clostridioides difficile* Strains

**DOI:** 10.3390/gels10120818

**Published:** 2024-12-12

**Authors:** Tomasz Bogiel, Patrycja Kwiecińska, Robert Górniak, Piotr Kanarek, Agnieszka Mikucka

**Affiliations:** 1 Microbiology Department, Ludwik Rydygier Collegium Medicum in Bydgoszcz, Nicolaus Copernicus University in Toruń, 9 Maria Skłodowska-Curie Street, 85-094 Bydgoszcz, Poland; patrycja.grochulska@onet.pl (P.K.); a.mikucka@cm.umk.pl (A.M.); 2 Microbiology Student Science Club, Ludwik Rydygier Collegium Medicum in Bydgoszcz, Nicolaus Copernicus University in Toruń, 9 Maria Skłodowska-Curie Street, 85-094 Bydgoszcz, Poland; robert.gorniak99@gmail.com; 3 Clinical Microbiology Laboratory, Dr. Antoni Jurasz University Hospital No. 1 in Bydgoszcz, 9 Maria Skłodowska-Curie Street, 85-094 Bydgoszcz, Poland; 4 Laboratory of Genetics and Molecular Biology, Prof. Dr. Stanisław Popowski Regional Specialized Children’s Hospital in Olsztyn, 18a Żołnierska Street, 10-561 Olsztyn, Poland; 5 Department of Microbiology and Food Technology, Faculty of Agriculture and Biotechnology, Bydgoszcz University of Science and Technology, 85-029 Bydgoszcz, Poland; piokan004@pbs.edu.pl

**Keywords:** agarose gel electrophoresis, antibiotic-associated diarrhea, binary toxin, *Clostridioides difficile*, *Clostridioides difficile* toxinotypes, multiplex PCR, toxin A, toxin B, toxinogenic potential

## Abstract

*Clostridioides difficile* is a common etiological factor of hospital infections, which, in extreme cases, can lead to the death of patients. Most strains belonging to this bacterium species synthesize very dangerous toxins: toxin A (TcdA) and B (TcdB) and binary toxin (CDT). The aim of this study was to assess the suitability of agarose gel electrophoresis separation of multiplex PCR amplicons to investigate the toxinogenic potential of *C. difficile* strains. Additionally, the frequency of *C. difficile* toxin genes and the genotypes of toxin-producing strains were determined. Ninety-nine *C. difficile* strains were used in the detection of the presence of genes encoding all of these toxins using the multiplex PCR method. In 85 (85.9%) strains, the presence of *tcdA* genes encoding enterotoxin A was detected. In turn, in 66 (66.7%) isolates, the gene encoding toxin B (*tcdB*) was present. The lowest number of strains tested was positive for genes encoding a binary toxin. Only 31 (31.3%) strains possessed the *cdtB* gene and 22 (22.2%) contained both genes for the binary toxin subunits (the *cdtB* and *cdtA* genes). A relatively large number of the strains tested had genes encoding toxins, whose presence may result in a severe course of disease. Therefore, the accurate diagnosis of patients, including the detection of all known *C. difficile* toxin genes, is very important. The multiplex PCR method allows for the quick and accurate determination of whether the tested strains of this bacterium contain toxin genes. Agarose gel electrophoresis is a useful tool for visualizing amplification products, allowing one to confirm the presence of specific *C. difficile* toxin genes as well as investigate their dissemination for epidemiological purposes.

## 1. Introduction

*Clostridioides difficile* is a strictly anaerobic, Gram-positive bacterium commonly found in soil and water. This bacillus was first identified in the feces of healthy infants and described in 1935 as *Bacillus difficilis*. Initially, these bacteria were thought to be the components of healthy human intestinal microbiota. However, it was not until 40 years later that they were identified, alongside other etiological factors, as a cause of the development of colitis associated with antibiotic therapy [1,2]. *C. difficile* infection typically occurs when bacterial spores enter the body from contaminated surfaces or other objects often touched by numerous individuals, e.g., toilets, telephones, doorknobs, handrails, etc. The bacteria can also be transmitted to patients via other people, so the proper hygiene of healthcare workers and visitors is essential, such as washing hands in warm water with soap and using disposable protective gloves. Additionally, the isolation of patients with diarrhea is required to prevent exposure and potential transmission to other individuals [3,4].

Toxins are the primary virulence factors of *C. difficile*. Most *C. difficile* strains produce two major protein toxins: TcdA and TcdB. These are encoded by the *tcdA* and *tcdB* genes, and classified within the cytotoxin family [5,6]. These toxins bind to cell membrane receptors and are subsequently internalized. Once in the cytosol, they inactivate guanosine-5′-triphosphate (GTP)-binding proteins of the Rho/Ras family. Members of the Ras protein superfamily consisting of Rho, Rac, and Cdc42 GTPases induce changes in intestinal cells, consisting of glycosylation caused by the mentioned toxins. This change leads to the activation of Rho protein guanosine triphosphate binding after the direct entry of toxins into the cytoplasm, resulting in the disruption of critical signaling pathways in the cell and the intracellular inactivation of GTPases. Additionally, toxins A and B can induce various morphological and physiological changes in intestinal epithelial cells [6]. Rho GTPases are also involved in numerous signaling processes, including the regulation of the actin cytoskeleton, cell polarization, gene transcription, and cell cycle progression. The alterations in the epithelial cell wall caused by Rho protein glycosylation involve at least two pathways. The disaggregation of actin microfilaments leads to increased tight junction permeability and the premature release of proinflammatory cytokines from the intestinal epithelium. This, in turn, stimulates mast, the vascular endothelium, and immune cells. The F-actin cytoskeleton forms aggregates after the cells change their shape [7].

Toxin A is a 308 kDa protein enterotoxin encoded by the *tcdA* gene. It induces hemorrhage, increased intestinal fluid secretion, and extensive damage to epithelial cells. Toxin A is regarded as the primary virulence factor of *C. difficile* due to its capacity to cause severe intestinal damage. It is primarily responsible for the intestinal symptoms observed in patients with *C. difficile* infection (CDI). Toxin A has been shown to disrupt the tight junctions of the intestinal epithelial lining, contributing to the mechanism of enterocytotoxicity. It plays a critical role in the pathogenesis of CDI by inducing cell rounding and detachment from the basement membrane, ultimately leading to apoptosis. This process results in the rapid loss of macrophages, T lymphocytes, and eosinophils. The severe inflammation driven by neutrophil infiltration leads to the denudation of the intestinal mucosa and damage to the intestinal epithelium [6,8].

Toxin B is a 269 kDa toxin encoded by the *tcdB* gene. This cytotoxin is nearly 1000 times more potent than toxin A. By binding to specific receptors on the intestinal membranes via toxin A, it contributes to their damage. This leads to the partial detachment of cells, and the formation of stress fibers, which, after filling the entire cell, disappear, and actin filaments accumulate in the perinuclear space. As a result of F-actin reorganization, a significant amount of potassium inside the cell is lost and the synthesis of proteins and nucleic acids is disrupted. Toxin B also inhibits the expression of interleukin-2, disrupts tight junctions between cells, and stimulates the production of nitric oxide [6,8].

Some strains of *C. difficile* also produce binary toxin, which is secreted as two distinct protein components: A (the enzymatic component) and B (the transport component). These components assemble on the host cell surface by binding the monomeric form of binary toxin subunit B (CDTb) to the lipolysis-stimulated lipoprotein receptor (LSR) [9]. CDTb then undergoes proteolytic activation and oligomerization on the cell surface. Alternatively, the monomeric form of CDTb may undergo proteolytic activation and oligomer formation before binding to the LSR. The oligomer mediates their binding to binary toxin subunit A (CDTa), and the receptor–binary toxin complex is then internalized into cells. Acidic pH triggers conformational changes in CDTb, leading to membrane disruption and the formation of a pore through which CDTa enters the cytosol. CDTa translocation is facilitated by heat shock proteins 70 (Hsp70) and 90 (Hsp90) and cyclophilin A (CypA). In the cytosol, the enzymatic component of the toxins, adenosine-5′-diphosphate (ADP), ribosylates G-actin on arginine-177, thereby blocking actin polymerization. Ultimately, the actin cytoskeleton disintegrates, cells round up, and microtubule-based protrusions form [10,11].

In this study, toxin genes were detected using multiplex polymerase chain reaction (PCR). This variant of the PCR method utilizes multiple pairs of different but gene-specific primers in one reaction, allowing for the simultaneous amplification of several DNA fragments [12]. The appropriate selection of primers that enables the amplification of products of different lengths allows for the detection of gene sequences encoding various products and, for example, the identification of several virulence factors of virulent strains. The primary advantage of the multiplex PCR method is the ability to simultaneously detect a larger number of DNA fragments while ensuring the accuracy of the process. This characteristic makes it particularly effective for use in microbiological diagnostics [13]. Following the amplification step, it is necessary to separate the amplified materials by electrophoresis, as in the case of classic PCR [12,14]. It is worth noting that gel electrophoresis is an established method used for the detection of genes encoding different properties among *C. difficile* strains [15,16].

Real-time PCR was used as a secondary diagnostic method in cases where the multiplex PCR method did not yield reliable and unequivocal results. This method is currently widely used in microbiological diagnostics to determine the number of genes and/or transcripts present in the tested material. The target specificity of any real-time PCR assay allows for the quantification of taxonomic or functional gene markers. Real-time PCR has also been shown to be an accurate, highly repeatable and sensitive technique for quantitatively tracking phylogenetic and functional gene changes [17,18]. Real-time PCR, like all PCR-based methods, is based on multiple amplification cycles. The DNA template is initially denatured, followed by the hybridization of oligonucleotide primers targeting specific sequences. The primers then extend along the complementary desired DNA fragment. Thermostable DNA polymerase activity leads to an exponential increase in the number of amplicons in subsequent reaction cycles. The main difference between real-time PCR and other PCR methods is the ability to assess the number of copies of amplicons during the reaction [17]. Real-time monitoring is enabled by the presence of, for example, fluorescent dyes or probes in the reaction [19].

The aim of this research was to simultaneously evaluate the presence of species-specific genes and *C. difficile* toxin genes, thereby identifying toxin-producing strains. Additionally, the study sought to determine the suitability of electrophoretic gel agarose separation of multiplex PCR products for assessing the toxigenic potential of *C. difficile* strains and its prevalence among the tested species.

## 2. Results and Discussion

### 2.1. Assessment of the Occurrence of Toxin-Encoding Genes Among C. difficile Strains

This research was carried out on 99 clinical strains and 2 control *C. difficile* strains. The aim of the study was to detect simultaneously the following toxin genes: *tcdA*, *tcdB*, *cdtA*, *cdtB*, the *gluD* gene, and the *16S rDNA* gene. Due to the possible contamination of the primers used for the detection of the *16S rDNA* gene fragment, they were excluded at a certain stage of the research.

To the best of our knowledge, agarose gel electrophoresis for multiplex PCR amplicon separation has been widely used so far but only for similar studies, not exactly for the approach applied in our study. Therefore, this is one of the first, or even the first, study focused on the application of agarose gel electrophoresis of multiplex PCR amplicon separation, exclusively targeting *C. difficile* toxins, in contrast to studies aiming at the detection of *C. difficile* along with other gastrointestinal infection pathogens or agarose gel-based *C. difficile* rybotyping or *C. difficile* rybotyping using multiplex PCR and subsequent capillary separation.

**Figure 1:**
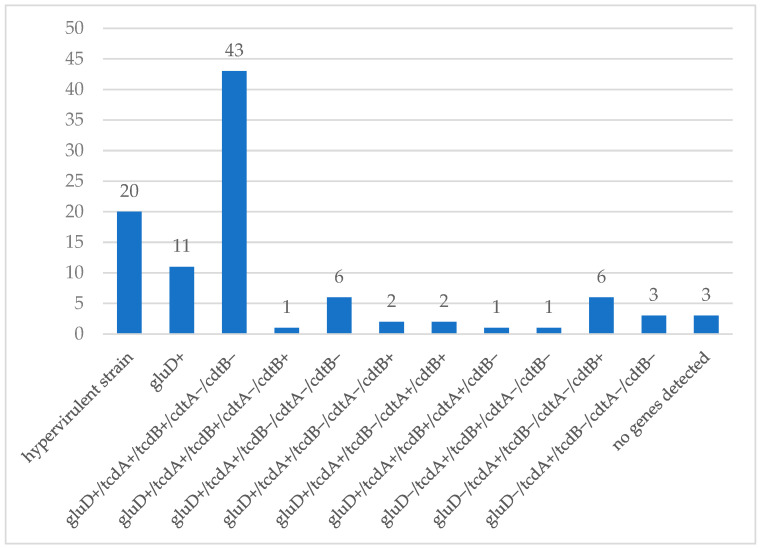
The diversity and number of the detected toxinogenotypes among *Clostridioides difficile* strains (*n* = 99), where *cdtA*+—presence of the gene encoding the A subunit of the binary toxin, *cdtA*−—no gene present, *cdtB*+—presence of the gene encoding the B subunit of the binary toxin, *cdtB*−—no gene present, *gluD*+—presence of the gene encoding glutamate dehydrogenase, *gluD*−—no gene present, *tcdA*+—presence of the gene encoding toxin A, *tcdA*−—no gene present, *tcdB*+—presence of the gene encoding toxin B, *tcdB*−—no gene present.

A statistical analysis of the occurrence of toxin-encoding genes was performed for 99 strains, in which a total of 291 gene fragments were detected. All five genes characteristic of hypervirulent strains were detected in 20 (20.2%) isolates. No fragments of the *tcdA*, *tcdB*, *cdtA,* or *cdtB* toxin genes were detected in 13 (13.1%) strains, and the *gluD* gene was also not detected in 3 strains. A graphical representation of the results obtained for the tested *C. difficile* isolates with respect to the particular toxino-genotype incidences is presented in Figure 1.

**Figure 2:**
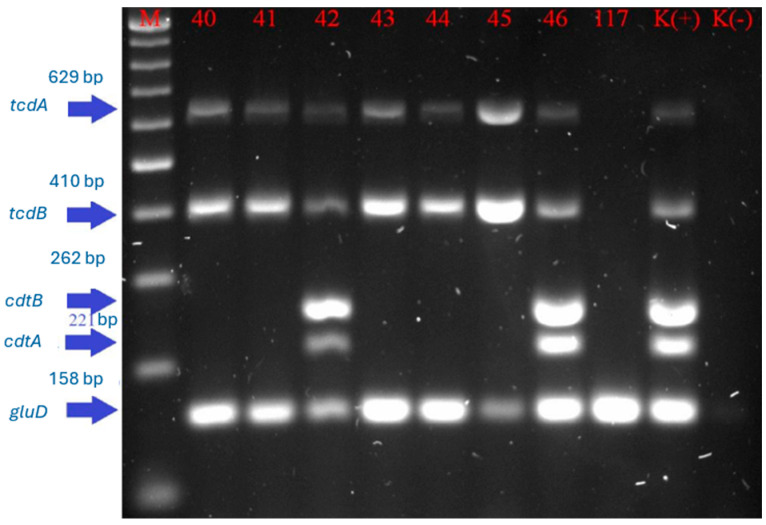
Example of agarose gel electrophoretic separation of the amplification products using the multiplex PCR technique for the *tcdA* (629 bp), *tcdB* (410 bp), *cdtB* (262 bp), *cdtA* (221 bp), and *gluD* (158 bp) genes, where *cdtA*—binary toxin subunit A gene; *cdtB*—binary toxin subunit B gene; *gluD*—glutamate dehydrogenase gene; *tcdA*—toxin A gene; *tcdB*—toxin B gene; M—DNA size marker 100–1000 base pairs; the lines labelled 40–46, 117—numbers of the tested strains; 42, 46, and K (+)—positive controls of the amplification reaction for all the investigated genes, and the remaining samples with the *gluD*, *tcdA,* and *tcdB* genes only; K (−)—negative control of the amplification reaction.

An example of the agarose gel electrophoretic separation of the amplicons is shown in Figure 2 for *C. difficile* samples numbered 40, 41, 42, 43, 44, 45, 46, and 117 (for details, see Supplementary Material, Table S1), which also includes a positive control (*C. difficile* control strain belonging to ribotype 027), a negative control (water), and a size marker to facilitate the interpretation of the agarose gel electrophoretic separation.

Despite our best efforts, for the strains numbered 52 to 116 (for details, see Supplementary Material, Table S1) the results of the multiplex PCR did not provide definitive and reliable results for the detection of the *tcdA* gene. Therefore, for this particular gene, and the mentioned strains, a second diagnostic method, the real-time PCR method, was applied and its results were considered as the final and most reliable ones.

### 2.2. Evaluation of Differences in the Frequency of Detected C. difficile Genes

A statistical analysis was performed using GraphPad Prism 9.3.1, applying one of the chi-square test variants. The statistical significance of the observed differences was assessed using this test, while for a comparison of the toxin genes with each other, the resulting *p* value was below < 0.0001. The detailed results obtained are presented in Table 1.

### 2.3. Correlation of the Occurrence of Toxin-Encoding Genes Among the Studied C. difficile Strains

The correlation value of the occurrence of toxin-encoding genes in the tested *C. difficile* strains was also evaluated. The analysis (Table 2) shows that the correlation coefficient is R = 0.736, which indicates a strong correlation between the occurrence of the *tcdA* and *tcdB* genes. Whenever the *tcdB* gene occurred (66.7%), the *tcdA* gene also appeared (85.9%); however, in the case of 19 (19.2%) strains, the *tcdB* gene was not detected, despite the presence of the *tcdA* gene.

Next, the correlation between the *tcdA*+ and the *cdtA*+ gene and the *cdtB*+ gene was examined. In both cases, correlation coefficients of R > 0.9 were obtained, indicating a very strong correlation. Whenever one or both binary toxin subunit-encoding genes were present, the *tcdA* gene was also detected. This means that the binary toxin-encoding gene does not occur without the toxin A gene, but the *tcdA* gene can occur independently of the binary toxin genes presence, as shown in Table 3 and Table 4.

In the next step, the correlation coefficient R was calculated for the gene encoding toxin B (*tcdB*) and the genes encoding the binary toxin subunits (*cdtA* and *cdtB*). In both cases, a high correlation was obtained.

For *tcdB* in relation to *cdtA*, the value of R = 0.82 was obtained, which indicates a high correlation of these genes’ presence. The gene encoding the binary toxin *cdtA* occurred in 23 strains, appearing as many as 21 times with the gene encoding toxin B. On the other hand, the correlation of the occurrence of the *tcdB* gene in relation to *cdtB* is R = 0.94, which indicates a very high correlation. The results obtained show that the *cdtB* gene is present in 31 (31.3%) of the strains studied, including as many as 24 (77.4%) strains with the presence of toxin B-encoding gene. The correlation results are presented in Table 5 and Table 6.

The last stage was the analysis of the correlation coefficient between both genes encoding the binary toxin, *cdtA* and *cdtB*. In this case, the correlation coefficient reached the value of R < 0.1. This shows that almost no statistically significant differences in the occurrence of these two genes were observed. The gene encoding the binary toxin *cdtA* occurs in 23.2% of the strains tested, while the *cdtB* gene occurs in 31.3% of them. Almost one-third of the strains tested containing the *cdtB* gene did not contain the *cdtA* gene. Only 4.5% of the strains containing the *cdtA* gene did not possess the *cdtB* gene. This indicates a much more frequent occurrence of the gene encoding the binary toxin subunits B (*cdtB*) than A (*cdtA*), which is presented in Table 7.

*C. difficile*, as a Gram-positive, anaerobic, spore-forming and toxin-producing bacillus, is a serious threat of hospital-associated infections worldwide [11,20]. Over the last decade, the incidence and severity of *C. difficile* infections have increased globally, establishing CDI as the most or one of the most common nosocomial infections [3]. Key factors contributing to the development of CDI include, e.g., exposure to antibiotics, advanced age, and hospitalization [11,21]. The clinical presentation of CDI is highly variable, ranging from asymptomatic carrier status, through to mild or moderate diarrhea, and in severe cases, to life-threatening fulminant colitis. Consequently, the development of a quick and effective diagnostic method for the detection of the toxin genes of *C. difficile* is of critical importance, as the toxins are responsible for most symptoms among infected individuals. To this end, some investigations were conducted using the multiplex PCR method to evaluate its effectiveness in detecting *C. difficile* toxin genes [22].

The existing literature addresses issues related to the spread of *C. difficile* strains [23,24,25]. However, a few studies have described the use of multiplex PCR methods for detecting toxin genes of this bacterium. In light of this, the results obtained in the current research were compared with those reported in the literature, which detail the detection of *C. difficile* toxin genes using not only the multiplex PCR method followed by agarose gel electrophoresis but also other diagnostic approaches.

Our research was conducted on 99 strains collected from patients in Poland. Four genes encoding *C. difficile* toxins were detected in the study. Using the multiplex PCR method with agarose gel amplicon separation, 20 (20.2%) hypervirulent strains were detected. A significantly higher number of hypervirulent strains was reported in research published in 2022 by Aptekorz et al. [26]. This study was conducted on 215 *C. difficile* strains, and toxinogenic genes, namely, those encoding TcdA, TcdB, and binary toxin, were detected in 180 (83.7%) of them. In other study published in 2021 by Kabała et al. [27], the authors identified 100% of the hypervirulent strains. The mentioned research was performed on 29 samples derived from Polish patients using a diagnostic method called Multiple-Locus Variable-Number Tandem Repeat Analysis (MLVA). In another analysis conducted between 2008 and 2018, published by Waker et al. [28], 140 *C. difficile* strains were detected, of which 122 (87.1%) were hypervirulent strains. The available data indicate that hypervirulent strains are increasingly being detected among Polish patients, which may lead to more severe disease symptoms and the faster spread of infections among hospitalized individuals.

In addition to hypervirulent strains, our research also identified other strains with the TcdA+/TcdB+ genotype, which accounted for 44 strains (44.4%) out of all the *C. difficile* isolates tested. Similar results were obtained in a 2007 study by Pituch et al. [29], where this profile was found in 80 tested isolates, representing 45.7% of all the identified strains. A much lower percentage of strains with this profile was obtained in the Waker et al. study [28], where the TcdA+/TcdB+ strains accounted for 6.4% only.

The available literature indicates that strains containing only toxin A are relatively rare. Despite this, our research revealed nine (9.1%) *C. difficile* strains with the TcdA+ genotype. A slightly lower percentage of strains with this genotype (3.7%) was obtained in the Aptekorz et al. [26] study. The summary of particular *C. difficile* strains’ toxinogenic potential described in the available literature is shown in Table 8.

In addition to the genetic profiles of *C. difficile* strains in the studies mentioned above, our own research identified the following genotypes: TcdA+/TcdB−/cdtA−/cdtB+ (eight; 8.1%); TcdA+/TcdB−/cdtA+/cdtB+ (two; 2.0%); TcdA+/TcdB+/cdtA+/cdtB− (one; 1.0%); TcdA+/TcdB+/cdtA−/cdtB+ (one; 1.0%). Nearly one-third (32; 32.3%) of the tested *C. difficile* isolates were identified as binary toxin-positive, with 12 (12.1%) of these not appearing hypervirulent. For comparison, in the study by Aptekorz et al. [26], one (0.5%) strain with a TcdA+/CDT+ profile was detected. Foreign research conducted by Azimirad et al. [30] showed the presence of genes encoding binary toxin in 21 out of 169 (12.4%) strains, when analyzed using the standard PCR method (Table 8).

The key gene indicating the presence of *C. difficile* bacteria is the *gluD* gene encoding glutamate dehydrogenase (GDH). Clinical samples with a negative GDH result are reported as *C. difficile*-negative. In our own research, as many as 13 strains that did not contain the GDH gene were determined. The lack of any investigated gene (in three samples) corresponds to a negative result of testing. Unfortunately, in the case of the 10 tested samples, toxins were detected despite the absence of the *gluD* gene. These results suggest a failure in the analysis, necessitating a re-examination of the samples with biological material. Such failure may be attributed to typical laboratory mistakes, e.g., the accidental omission of one of the necessary primers or the addition of an insufficient volume. Another potential cause could be the inadequate pipetting of the reaction mixture. To address this, these strains should be re-tested using the monoplex or real-time PCR method or another diagnostic approach.

The majority of existing studies focus on research conducted globally, rather than for patients of Polish hospitals. One of the available works is a study conducted by Azimirad et al. [30] regarding the analysis of 169 *C. difficile* strains obtained from biological samples taken from Iranian patients. The isolates were tested using the PCR method to detect the *tcdA*, *tcdB*, *cdtA,* and *cdtB* genes. The analysis showed that 144 (85.2%) of the tested strains were recognized as samples with a positive result for *C. difficile* presence. Their genotyping revealed that 124 of 144 (86.1%) toxigenic strains contained two toxins: TcdA and TcdB. Only 11 (7.6%) strains had the genotype indicating the presence of toxin A only. Our own research detected a similar percentage of strains with toxin-encoding genes (85.9%) and 44.4% belonged to the *tcdA*+/*tcdB*+ genotype (Table 8).

Based on the results of research by Heidari et al. [31] on 45 toxigenic *C. difficile* isolates, it was found that the *tcdA*+/*tcdB*+ genotype, as the most common toxicity profile, was found among 37 (82.2%) isolates. However, only one isolate was identified as a hypervirulent strain, while at least one of the binary toxin-encoding gene subunits was detected in seven (15.5%) isolates. Similar results were obtained in a study conducted by Tokimatsu et al. [32]. They showed the presence of genes for both toxins (*tcdA* and *tcdB*) in 135 (80.8%) isolates. In the case of 23 tested strains (13.8%), the presence of the toxin B gene only was confirmed. The occurrence of all toxin-coding genes was observed in nine (5.4%) of the tested isolates. In our own research, no strains with the *tcdA*−/*tcdB*+ genotype were observed. The presence of toxin B was always correlated with the presence of toxin A. No *tcdA*−/*tcdB*+ genotypes were detected in the studies conducted by Deniz et al. [33] (Table 8).

The differences between the results of foreign research and the results of our own study and these conducted in Poland by other researchers may be attributed to several factors, including the current epidemiological situation, localized outbreaks of specific infections, the dissemination of local strains, variations in cultural background, differences in diet, and the use of distinct pharmacological treatment methods during hospitalization.

After completing the current study, the results obtained from the *C. difficile* isolates were compared with pilot studies conducted in 2019 at the Department of Microbiology of the Ludwik Rydygier Collegium Medicum in Bydgoszcz Nicolaus Copernicus University in Toruń. These earlier studies utilized conventional PCR techniques, whereas our research employed multiplex PCR methods. Their analysis using standard PCR revealed that nearly 86.8% of the tested strains exhibited the presence of toxin-encoding genes, and a comparable result (85.9%) was obtained in the current study. The most notable difference is observed in the percentage of detected genes encoding toxin B. The study conducted in 2019 indicated that nearly 86.8% of the strains exhibited the presence of the toxin B-encoding gene, whereas our research found that this gene was present in only 66.6% of the isolates. The gene encoding toxin A was detected in 86.8% of the strains tested in 2019, and a similar result (85.9%) was obtained in our own studies. The binary toxin gene was detected in 43.4% of the isolates using the standard PCR method, while using the multiplex method, it was detected in only 32.3%. It is worth noting that these percentage differences may depend on the number of strains tested as well as the differences in the PCR primer sequences. The 2019 study was conducted on 106 *C. difficile* isolates, while in our own study, 99 strains were used. In the quoted study, the gene encoding toxin B was not detected in eight isolates, and also not in the control sample. However, the tests were repeated, and for unknown reasons, the detected genes ceased to be amplified and as a result, were not visible on the agarose gel, which may indicate a false negative result of our own tests. Previous studies by the current researchers have also highlighted the effectiveness and usefulness of using separation with classic and capillary gel electrophoresis [34,35].

A review of the literature along with an analysis of our own results, regarding the frequency of genes encoding *C. difficile* toxins, clearly demonstrates that the majority of strains possess genes determining toxigenicity. The *tcdA* and *tcdB* genes are detected much more frequently than *cdt* (*cdtA*, *cdtB*).

In summary, multiplex PCR analysis accompanied with agarose gel electrophoresis-based amplicon separation can be a reliable diagnostic method for detecting strains containing *C. difficile* toxin genes. In addition, the simple methodology, low cost, and easy interpretation criteria allow this method to be used in almost every diagnostic laboratory.

## 3. Conclusions

The multiplex PCR technique is a relatively simple diagnostic method that enables the simultaneous detection of individual *C. difficile* toxin genes. Most *C. difficile* strains (86.9%) exhibit toxigenic potential, highlighting the importance of the rapid and reliable diagnosis of *C. difficile* infections. The prevalence of the *tcdA* and *tcdB* genes in most strains suggests that the TcdA and TcdB toxins may be more prevalent than the binary toxin. In the context of assessing the frequency of toxin genes and their genotypes of toxigenic potential of strains, agarose gel electrophoresis complements the multiplex PCR method, providing a quick and accurate means of identifying hypervirulent *C. difficile* strains.

## 4. Materials and Methods

### 4.1. Strains Tested

The research included 99 *C. difficile* strains, obtained from the collection of Microbiology Department Ludwik Rydygier Collegium Medicum in Bydgoszcz Nicolaus Copernicus University in Toruń, Poland. The strains were isolated from diarrheal stool samples collected from patients hospitalized at Dr. Antoni Jurasz University Hospital No. 1. in Bydgoszcz, Poland.

### 4.2. DNA Isolation

Isolation of genomic DNA of the tested and reference strains was performed according to the manufacturer’s instructions. The EXTRACTME^®^ DNA BACTERIA KIT was used for this purpose (Blirt, DNA GDAŃSK, Gdańsk, Poland).

Briefly, the *C. difficile* colonies obtained on Columbia Agar with 5% sheep blood (*bio*Mérieux, Marcy-l’Étoile, France), on which biological material was previously cultured anaerobically, have been used for further research. The colonies were sampled using a sterile loop and then suspended in 300 μL of lysis buffer for cell lysis. To eliminate ribonucleic acid (RNA), 4 μL of RNase A (SIGMA) and 40 μL of lysozyme (SIGMA) at a concentration of 100 mg/mL were added to improve DNA isolation. Samples were vortexed and placed in a Thermomixer comfort thermal cycler (Eppendorf, Hamburg, Germany). The samples were incubated at 37 °C for 30 min. In the next stage, proteinase K was added at a volume of 10 μL to clean up the material from protein compounds. The samples were then incubated in a thermocycler at 55 °C for 10 min. Following this, the samples were vortexed and incubated again in the thermal cycler at 55 °C for an additional 5 min. After this step, the samples were vortexed for 15 s and then centrifuged at 13,000× *g* for 2 min in a MiniSpin^®^ plus centrifuge (Eppendorf, Hamburg, Germany). After the step of sample centrifugation, the supernatant was collected and purified using mini silica columns. The samples placed on columns were centrifuged again at 13,000× *g* for one minute. Then, 600 μL of BacW wash buffer was added to the mini columns placed on the new collection tubes and the samples were centrifuged at 13,000× *g* for 30 s. The receiving tubes were emptied and mini columns placed on top of them again. Then, BacW wash buffer was added at a volume of 400 μL. The samples were centrifuged again at 13,000× *g* for 30 s. The collection tubes were again emptied and the mini columns placed in them, following material centrifugation at 14,000× *g* for 2 min. Mini columns were placed in sterile 1.5 mL Eppendorf tubes. An elution buffer, previously heated to 70 °C, was added at a volume of 80 μL. Next, the samples were incubated at room temperature for two minutes. After the incubation of samples, they were centrifuged again at 13,000× *g* for one minute to elute the nucleic acid. The mini columns were then removed. Samples prepared in this way were kept until analysis at 4 °C, or in the case of longer storage, at −20 °C. In the final step, the effectiveness of genomic DNA isolation was assessed using BioPhotometer^®^ D30 (Eppendorf, Hamburg, Germany), with spectrophotometric measurements at 260 and 280 nm wavelengths to confirm their quality and appropriateness for the research.

### 4.3. Detection of C. difficile Toxin Genes

Multiplex PCR was employed to detect *C. difficile* species-specific and toxin genes. A real-time PCR, considered a more sensitive diagnostic method, was applied for verification of the results obtained from some of the samples. This approach aimed at clarification of the obtained results. After amplification using multiplex PCR, it was necessary to visualize the results, and for this purpose, electrophoretic separation in a 2.5% agarose gel was used.

#### 4.3.1. Multiplex PCR Technique

The multiplex PCR technique utilized genomic DNA isolated from the tested and the reference (*C. difficile* ribotype 027) strains, with the confirmed presence of toxin A, toxin B, and binary toxin genes, treated as a positive control. The negative control was molecular biology-grade water (Eppendorf). All the reagents in appropriate volumes (Table 9), water, primers (Sigma, Kawasaki, Japan), and Hot Start Master Mix (Qiagen, Hilden, Germany), were placed in a sterile Eppendorf tube stored on ice, in sterile conditions, under a laminar chamber in order to reduce the risk of contamination. The reaction mixture was added to PCR tubes at a volume of 22.5 μL, and then either 2.5 μL of isolated DNA of the tested strains was added or *C. difficile* reference strain, or water. The list of volumes of reagents used for the multiplex PCR is presented in Table 9. In Table 10, the specification of primers used in this research is presented in detail, according to European Centre for Disease Prevention and Control Recommendations (https://www.ecdc.europa.eu/en/clostridioides-difficile-infections, accessed on 17 July 2024).

The multiplex PCR was performed in a Mastercycler^®^ pro (Eppendorf, Hamburg, Germany) to detect the following genes: *tcdA*, *tcdB*, *cdtA*, *cdtB*, *16 S-rDNA* and *GluD*. This was performed with initial denaturation at 94 °C for 15 min, followed by 35 cycles consisting of denaturation at 94 °C for 15 s, primer annealing at 50 °C for 45 s, primer extension at 72 °C for 1 min, and a single step of the final elongation at 72 °C for 30 min.

#### 4.3.2. Agarose Gel Electrophoresis for Multiplex PCR Amplicon Separation

After completing gene amplification using the multiplex PCR method, agarose gel electrophoresis-based separation was performed. The agarose gel was prepared by adding 2.5 g of agarose (Blirt^®^, Gdańsk, Poland), 10 mL of 10× TBE (Tris-Boric Acid-EDTA) buffer (BioRad^®^, Hercules, CA, USA), 6 µL of Midori Green intercalating dye (NIPPON Genetics EUROPE, GmbH, Düren, Germany), and 90 µL of distilled water. All the ingredients were boiled to dissolve the agarose, then cooled to ambient temperature and poured over into agarose gel stand to allow it to solidify at room temperature. This resulted in a composition of 2.5% agarose gel in 1x TBE buffer (BioRad^®^).

Amplicons at volumes of 8 μL were transferred to each well of the prepared agarose gel. Apart from for the product amplification, 6 μL of Perfect Size Marker (100–1000 bp DNA Ladder, Eurx) was also placed in separate wells of the gel to assess the size of the obtained DNA fragments. Electrophoresis was performed using a PowerPac™ Basic electrophoresis apparatus (BioRad^®^, Feldkirchen, Germany) under the following conditions: voltage—60 V, current—400 mA, time—120 min. Visualization was performed under UV light using a camera and GelDoc XR+ software Imaging System (BioRad^®^, Feldkirchen, Germany). The interpretation of the electrophoretic separation results was based on the assessment and comparison of the presence and sizes of the obtained bands with respect to the control strains.

#### 4.3.3. Real-Time PCR Technique

The real-time PCR technique was used for samples in which the *tcdA* gene could not be detected in either the tested or control strains. The control sample was the genomic DNA isolated from the *C. difficile* strain, the same as used in the multiplex method. Reactions were performed for the *tcdA* gene on real-time PCR optical plates (Roche, Rotkreuz, Switzerland), in a final volume of 20 μL. The reaction mixture was prepared in a sterile Eppendorf tube (Eppendorf, Hamburg, Germany), placed on ice, under sterile conditions, in the chamber laminar to avoid contamination. A volume of 19 μL of the mixture was added to each well on the plate and 1 μL of isolated DNA/water was added subsequently. A summary of the concentrations and volumes of the reagents used is presented in Table 11. The primers used for the reaction are the same as those used for the multiplex PCR to detect the *tcdA* gene.

Real-time PCRs were performed on the CFX Opus 96 Dx Real-Time PCR System (BioRad^®,^, Feldkirchen, Germany). The following amplification reaction conditions were used for the detection of the *tcdA* gene: initial denaturation at 95 °C for 12 min, followed by 50 cycles each consisting of denaturation at 95 °C for 15 s, primer annealing and DNA amplification at 65 °C for 20 s. To confirm the amplification specificity, the analysis of the melting curve of the amplification product was performed using the high-resolution melting technique, with a temperature increase from 65 °C to 97 °C, increments of 0.5 °C every 5 s, and constant read-outs. The entire reaction took about two hours.

### 4.4. Statistical Analysis Methods

Statistical analysis was performed in two statistical programs. Excel (Microsoft Office, v. 2021) and GraphPad Prism 9.0.0 (Dotmatics, Boston, MA, USA) were used, by verifying the obtained results based on a significance coefficient of α = 0.05. Such settings allowed us to consider all differences occurring that were statistically significant at the test probability of *p* < 0.05.

## Figures and Tables

**Table 1:** Comparison of the level of statistical significance in the analysis of differences in the frequency of toxin-encoding genes in the studied *C. difficile* strains (*n* = 99).

Gene	*tcdA*	*tcdB*	*cdtA*	*cdtB*	*gluD*	*p* Value
Presence	85	66	23	31	86	<0.0001
Absence	14	33	76	65	13	

*cdtA*—binary toxin subunit A gene, *cdtB*—binary toxin subunit B gene, *gluD*—glutamate dehydrogenase gene, *tcdA*—toxin A gene, *tcdB*—toxin B gene.

**Table 2:** Assessment of the presence of the *tcdA* gene in relation to the *tcdB* gene.

Determining the Presence of *tcdA*		Determining the Presence of *tcdB*		Correlation Coefficient R
		tcdB+	tcdB−	
*tcdA*+	*n*	66	19	0.74
	%	77.6%	22.4%	
*tcdA*−	*n*	0	14	
	%	0.0%	100.0%	

*tcdA*+—toxin A gene present, *tcdA*−—toxin A gene absent, *tcdB*+—toxin B gene present, *tcdB*−—toxin B gene absent, *n*—the number of strains with a particular toxin-encoding gene.

**Table 3:** Assessment of the presence of the *tcdA* gene corresponding to the *cdtA* gene.

Determining the Presence of tcdA		Determining the Presence of cdtA		Correlation Coefficient R
		*cdtA*+	*cdtA*−	
*tcdA*+	*n*	23	62	0.98
	%	27.1%	72.9%	
*tcdA*−	*n*	0	14	
	%	0.0%	100.0%	

*cdtA*+—binary toxin gene present, *cdtA*−—binary toxin gene absent, *tcdA*+—toxin A gene present, *tcdA*−—toxin A gene absent, *n*—number of strains with a given toxin gene.

**Table 4:** Assessment of the presence of the *tcdA* gene with respect to the *cdtB* gene.

Determining the Presence of *tcdA*		Determining the Presence of cdtB		Correlation Coefficient R
		*cdtB*+	*cdtB*−	
*tcdA*+	*n*	31	54	0.97
	%	36.5%	63.5%	
*tcdA*−	*n*	0	14	
	%	0.0%	100.0%	

*cdtB*+—binary toxin gene present, *cdtB*−—binary toxin gene absent, *tcdA*+—toxin A gene present, *tcdA*−—toxin A gene absent, *n*—number of strains with a given gene.

**Table 5:** Assessment of the presence of the *tcdB* gene in relation to the *cdtA* gene.

Determining the Presence of *tcdB*		Determining the Presence of *cdtA*		Correlation Coefficient R
		cdtA+	cdtA−	
*tcdB*+	*n*	21	45	0.819
	%	31.8%	68.2%	
*tcdB*−	*n*	2	31	
	%	6.1%	93.9%	

*cdtA*+—binary toxin gene present, *cdtA*−—binary toxin gene absent, *tcdB*+—toxin B gene present, *tcdB*−—toxin B gene absent, *n*—number of strains with a given gene.

**Table 6:** Assessment of the presence of the *tcdB* gene with respect to the *cdtB* gene.

Determining the Presence of *tcdB*		Determining the Presence of *cdtA*		Correlation Coefficient R
		*cdtB*+	*cdtB*−	
*tcdB*+	*n*	24	42	0.942
	%	36.4%	63.6%	
*tcdB*−	*n*	7	26	
	%	21.2%	78.8%	

*cdtB*+—binary toxin gene present, *cdtB*−—binary toxin gene absent, *tcdB*+—toxin B gene present, *tcdB*−—toxin B gene absent, *n*—number of strains with a given gene.

**Table 7:** Assessment of the presence of the *cdtA* gene relative to the *cdtB* gene.

Determining the Presence of *cdtA*		Determining the Presence of cdtB		Correlation Coefficient R
		cdtB+	cdtB−	
*cdtA*+	n	22	1	0.076
	%	70.97%	3.23%	
*cdtA*−	n	9	53	
	%	13.24%	98.53%	

*cdtA*+—gene encoding binary toxin present, *cdtA*−—gene encoding toxin B absent, *cdtB*+—gene encoding binary toxin present, *cdtB*−—gene encoding binary toxin absent, *n*—number of strains with a given gene.

**Table 8:** The presence of toxinogenic potential described previously in the available literature for particular *C. difficile* strains, numbered with respect to the corresponding research.

*C. difficile* Toxinogenic Potential [%]					*n*	Reference
tcdA Only	tcdB Only	tcdA/tcdB Only	cdt Genes	tcdA/tcdB/cdt		
9.1	0.0	44.4	32.3	22.2	99	This study
3.7	0.0	10.7	84.2	83.7	215	Aptekorz et al. [26]
0.0	0.0	0.0	100.0	100.0	29	Kabała et al. [27]
0.0	0.0	6.4	87.1	87.1	140	Waker et al. [28]
0.0	30.3	45.7	8.6	8.6	175	Pituch et al. [29]
4.7	4.7	73.4	12.4	8.2	169	Azimirad et al. [30]
0.0	4.5	82.2	15.5	2.2	45	Heidari et al. [31]
0.0	13.8	80.8	5.4	5.4	167	Tokimatsu et al. [32]
0.0	0.0	72.0	0.0	0.0	50	Deniz et al. [33]

*cdt* genes—binary toxin genes present, *tcdA*—toxin A gene present only, *tcdB*—toxin B gene present only, *tcdA/tcdB*—toxin A and B genes present simultaneously, *tcdA/tcdB/cdt*—toxin A, B, and binary toxin genes present simultaneously, *n*—number of samples included in particular cited study.

**Table 9:** Summary of volumes and concentrations of reagents used for preparation reaction mixture used in the multiplex PCR method.

Reagent	Volume per Sample [μL]	Initial Concentration of Reagents
PCR-grade water (Eurx)	6.5	-
Hot Start Master Mix (Qiagen)	12.5	5 U/μL
Primer tcdA-F (50× diluted)	1	100 μM
Primer tcdA-R(50× diluted)	1	100 μM
Primer tcdB-F (50× diluted)	0.4	100 μM
Primer tcdB-RA (50× diluted)	0.2	100 μM
Primer tcdB-RB (50× diluted)	0.2	100 μM
Primer cdtA-FA (50× diluted)	0.05	100 μM
Primer cdtA-FB (50× diluted)	0.05	100 μM
Primer cdtA-R (50× diluted)	0.1	100 μM
Primer cdtB-F (50× diluted)	0.1	100 μM
Primer cdtB-R (50× diluted)	0.1	100 μM
Primer PS-F (50× diluted)	0.05	100 μM
Primer PS-R (50× diluted)	0.05	100 μM
Primer GluD-F (50× diluted)	0.1	100 μM
Primer GluD-R (50× diluted)	0.1	100 μM

**Table 10:** Specification of the primers (Sigma) used for multiplex PCR tests.

Name	Gene	Primer Sequence 5′→3′	Amplicon Size [bp]
tcdA-F	*tcdA*	5′-GCATGATAAGGCAACTTCAGTGGTA-3′	629
tcdA-R		5′-AGTTCCTCCTGCTCCATCAAATG-3′	
tcdB-F	*tcdB*	5′-CCAAARTGGAGTGTTACAAACAGGTG-3′	410
tcdB-RA		5′-GCATTTCTCCATTCTCAGCAAAGTA-3′	
tcdB-RB		5′-GCATTTCTCCGTTTTCAGCAAAGTA-3′	
cdtA-FA	*cdtA*	5′-GGGAAGCACTATATTAAAGCAGAAGC-3′	221
cdtA-FB		5′-GGGAAACATTATATTAAAGCAGAAGC-3′	
cdtA-R		5′-CTGGGTTAGGATTATTTACTGGACCA-3′	
cdtB-F	*cdtB*	5′-TTGACCCAAAGTTGATGTCTGATTG-3′	262
cdtB-R		5′-CGGATCTCTTGCTTCAGTCTTTATAG-3′	
PS-F	*16S-rDNA*	5′-GGAGGCAGCAGTGGGGAATA-3′	1062
PS-R		5′-TGACGGGCGGTGTGTACAAG-3′	
GluD-F	*gluD*	5′-GTCTTGGATGGTTGATGAGTAC-3′	158
GluD-R		5′-TTCCTAATTTAGCAGCAGCAGCTTC-3′	

bp—base pairs, cdtA—binary toxin subunit A gene, cdtB—binary toxin subunit B gene, gluD—glutamate dehydrogenase gene, tcdA—toxin A gene, tcdB—toxin B gene.

**Table 11:** Summary of the reagents used in real-time PCR: volumes and initial and final concentrations.

Reagent	Volume Per Sample	Final Concentration
5× HOT FIREPol^®^ EvaGreen^®^ qPCR Mix Plus (SolisBiodyne)	4 μL	1×
TcdA-F (50× dilution)	2.5 μL	250 nM
TcdA-R (50× dilution)	2.5 μL	250 nM
PCR-grade water	10 μL	-
DNA	1 μL	-
Total	19 μL	-

## Data Availability

The raw data supporting the conclusions of this article will be made available by the authors on request.

## Supplementary Materials

The following supporting information can be downloaded at https://www.mdpi.com/article/10.3390/gels10120818/s1: Table S1: The detailed results of toxinogenic gene detection for the investigated *Clostridioides difficile* strains.
