# Supplementary material for: The Use of Gel Electrophoresis to Separate Multiplex Polymerase Chain Reaction Amplicons Allows for the Easy Identification and Assessment of the Spread of Toxigenic Clostridioides difficile Strains
Source: Gels. 2024 Dec 12;10(12):818. doi: 10.3390/gels10120818 (PMC11675878; doi:10.3390/gels10120818)
Supplement: Supplementary file 1 [file gels-10-00818-s001.zip › gels-3333411-supplementary.pdf]

gels

Supplementary material

Article

The use of gel electrophoresis to separate multiplex PCR amplicons allows for easy identification and assessment of the spread of toxigenic *Clostridioides difficile* strains

Tomasz Bogiel [1,2,3,*,†], Patrycja Kwiecińska[1,†], Robert Górniak [2,4], Piotr Kanarek [5] and Agnieszka Mikucka [1,3]

[1] Microbiology Department, Ludwik Rydygier Collegium Medicum in Bydgoszcz, Nicolaus Copernicus University in Toruń, 9 Maria Skłodowska-Curie Street, 85-094 Bydgoszcz, Poland, t.bogiel@cm.umk.pl (T.B.), patrycja.grochulska@onet.pl (P.K.), a.mikucka@cm.umk.pl (A.M.)
[2] Microbiology Student Science Club, Ludwik Rydygier Collegium Medicum in Bydgoszcz, Nicolaus Copernicus University in Toruń, 9 Maria Skłodowska-Curie Street, 85-094 Bydgoszcz, Poland, robert.gorniak99@gmail.com (R.G.)
[3] Clinical Microbiology Laboratory, Dr. Antoni Jurasz University Hospital No. 1 in Bydgoszcz, 9 Maria Skłodowska-Curie Street, 85-094 Bydgoszcz, Poland
[4] Laboratory of Genetics and Molecular Biology, Laboratory of Genetics and Molecular Biology, Prof. Dr. Stanisław Popowski Regional Specialized Children's Hospital in Olsztyn, 18a Żołnierska Street, 10-561 Olsztyn, Poland
[5] Department of Microbiology and Food Technology, Faculty of Agriculture and Biotechnology, Bydgoszcz University of Science and Technology, 85-029 Bydgoszcz, Poland, piokan004@pbs.edu.pl (P.K.)
* Correspondence: t.bogiel@cm.umk.pl, Tel.: +48 52 585 44 80
† Authors contributed equally to this publication

Table S1. The detailed results of toxinogenic genes detection for the investigated *Clostridioides difficile* strains (n = 99)

Strain No.	*GluD*	*cdtA*	*cdtB*	*tcdB*	*tcdA*	Strain No.	*GluD*	*cdtA*	*cdtB*	*tcdB*	*tcdA*	Strain No.	*GluD*	*cdtA*	*cdtB*	*tcdB*	*tcdA*
1	+	-	-	+	+	40	+	-	-	+	+	76	+	-	-	-	+
3	+	-	-	+	+	41	+	-	-	+	+	77	-	-	+	-	+
4	+	-	-	+	+	42	+	+	+	+	+	78	-	-	+	-	+
5	+	-	-	+	+	43	+	-	-	+	+	79	+	+	+	+	+
6	+	-	-	+	+	44	+	-	-	+	+	80	+	-	-	+	+
7	+	-	-	+	+	45	+	-	-	+	+	81	+	-	-	+	+
8	+	-	-	+	+	46	+	+	+	+	+	82	+	+	-	+	+
9	+	-	-	+	+	47	+	-	-	-	-	84	-	-	-	-	+
10	+	-	-	+	+	48	+	-	-	-	-	86	+	-	-	+	+
12	+	-	-	+	+	49	+	-	-	+	+	90	+	+	+	+	+
13	+	+	+	+	+	50	+	-	-	+	+	91	+	+	+	+	+
14	+	-	-	+	+	51	+	-	-	-	-	92	-	-	-	-	+
15	+	+	+	+	+	52	+	-	-	-	-	93	+	+	+	+	+
16	+	-	-	+	+	53	+	-	-	+	+	101	+	+	+	+	+
17	+	-	-	-	-	54	+	-	-	-	-	102	+	-	-	+	+
18	+	-	+	+	+	55	+	-	-	-	-	103	-	-	+	-	+
19	+	-	-	-	+	56	+	+	+	+	+	104	-	-	+	-	+
21	+	-	+	-	+	57	+	-	-	+	+	105	+	+	+	+	+
22	+	-	-	-	+	58	-	-	-	-	-	106	-	-	+	-	+
23	+	-	-	+	+	59	+	-	-	+	+	107	+	+	+	+	+
24	+	+	+	+	+	60	+	-	-	+	+	108	+	+	+	+	+
25	+	+	+	+	+	62	+	+	+	+	+	109	+	-	-	+	+
26	+	-	-	+	+	63	+	+	+	+	+	117	+	-	-	-	-
27	-	-	-	-	-	64	+	-	-	+	+	118	+	+	+	-	+

28	+	-	-	-	-	66	+	+	+	+	+	119	+	+	+	-	+
30	+	-	-	+	+	67	+	-	-	+	+	120	-	-	-	-	-
32	+	-	-	+	+	68	+	+	+	+	+	121	+	-	+	-	+
33	+	-	-	+	+	69	+	-	-	+	+	122	+	-	-	-	+
34	+	-	-	+	+	70	+	-	-	-	-	123	+	-	-	-	+
36	+	-	-	+	+	71	-	-	+	-	+	125	+	-	-	-	+
37	+	-	-	+	+	72	+	-	-	+	+	126	-	-	-	-	+
38	+	-	-	+	+	73	+	-	-	-	-	127	+	-	-	+	+
39	+	+	+	+	+	74	+	-	-	+	+	128	-	-	-	+	+

cdtA – binary toxin subunit A gene, *cdtB* – binary toxin subunit B gene, *gluD* – glutamate dehydrogenase gene, *tcdA* – toxin A gene, *tcdB* – toxin B gene, + – presence of a particular gene, - – absence of a particular gene
